# Effect of Early vs. Delayed or No Intubation on Clinical Outcomes of Patients With COVID-19: An Observational Study

**DOI:** 10.3389/fmed.2020.614152

**Published:** 2020-12-23

**Authors:** Ilias I. Siempos, Eleni Xourgia, Theodora K. Ntaidou, Dimitris Zervakis, Eleni E. Magira, Anastasia Kotanidou, Christina Routsi, Spyros G. Zakynthinos

**Affiliations:** ^1^First Department of Critical Care Medicine and Pulmonary Services, Evangelismos Hospital, National and Kapodistrian University of Athens Medical School, Athens, Greece; ^2^Division of Pulmonary and Critical Care Medicine, Department of Medicine, New York-Presbyterian Hospital-Weill Cornell Medical Center, Weill Cornell Medicine, New York, NY, United States

**Keywords:** acute respiratory distress syndrome, ARDS, acute respiratory failure, coronavirus, viral pneumonia, critically ill

## Abstract

**Background:** Optimal timing of initiation of invasive mechanical ventilation in patients with acute hypoxemic respiratory failure due to COVID-19 is unknown. Thanks to early flattening of the epidemiological curve, ventilator demand in Greece was kept lower than supply throughout the pandemic, allowing for unbiased comparison of the outcomes of patients undergoing early intubation vs. delayed or no intubation.

**Methods:** We conducted an observational study including all adult patients with laboratory-confirmed COVID-19 consecutively admitted in Evangelismos Hospital, Athens, Greece between March 11, 2020 and April 15, 2020. Patients subsequently admitted in the intensive care unit (ICU) were categorized into the “early intubation” vs. the “delayed or no intubation” group. The “delayed or no intubation” group included patients receiving non-rebreather mask for equal to or more than 24 h or high-flow nasal oxygen for any period of time or non-invasive mechanical ventilation for any period of time in an attempt to avoid intubation. The remaining intubated patients comprised the “early intubation” group.

**Results:** During the study period, a total of 101 patients (37% female, median age 65 years) were admitted in the hospital. Fifty-nine patients (58% of the entire cohort) were exclusively hospitalized in general wards with a mortality of 3% and median length of stay of 7 days. Forty-two patients (19% female, median age 65 years) were admitted in the ICU; all with acute hypoxemic respiratory failure. Of those admitted in the ICU, 62% had at least one comorbidity and 14% were never intubated. Early intubation was not associated with higher ICU-mortality (21 vs. 33%), fewer ventilator-free days (3 vs. 2 days) or fewer ICU-free days than delayed or no intubation.

**Conclusions:** A strategy of early intubation was not associated with worse clinical outcomes compared to delayed or no intubation. Given that early intubation may presumably reduce virus aerosolization, these results may justify further research with a randomized controlled trial.

## Background

Management of acute hypoxemic respiratory failure associated with coronavirus disease 2019 (COVID-19) often includes mechanical ventilation (1, 2). Optimal timing of initiation of invasive mechanical ventilation remains unknown. On the one hand, early initiation of invasive mechanical ventilation (i.e., early endotracheal intubation) has been advocated to avoid alternate means of oxygenation (such as high-flow nasal oxygen or non-invasive mechanical ventilation) associated with aerosolization of virus (3). Also, early intubation may prevent induction of harmful self-inflicting lung injury in patients who breath spontaneously and have large transpulmonary pressure swings (4). On the other hand, skeptics of early intubation may retort that intubation itself may generate viral aerosols (5), while the concept of self-inflicting lung injury (which could presumably be prevented by early intubation) may not yet be sufficiently supported by solid scientific data (6, 7). Furthermore, delaying intubation, by trying alternate means of oxygenation, may mean that some patients may not be intubated at all and therefore will be protected from the adverse events of invasive mechanical ventilation. The latter strategy may also address the shortage of ventilators to meet the increased demand of treating patients with COVID-19.

Ventilator supply-demand mismatch could have affected clinical decision-making regarding application of early vs. delayed or no intubation in several epicenters of the pandemic (8), i.e., the possibility could not be precluded that physicians might be forced not to intubate as part of a triage if ventilators were missing. Accordingly, ventilator supply-demand mismatch could also have affected clinical outcomes (9) and may therefore have acted as a confounder when attempting to estimate the effect of early vs. delayed or no intubation on clinical outcomes of patients with COVID-19 in several epicenters of the pandemic. This might not be the case for Greece where early implementation of social distancing measures and flattening of the epidemiological curve reduced burden of health-care system, constantly maintaining ventilator demand lower than supply. This fact allowed for an unbiased estimation as to whether early intubation as opposed to delayed or no intubation affects prognosis of patients with COVID-19. We hypothesized that early intubation is not associated with worse clinical outcomes, including mortality, than delayed or no intubation among patients with acute hypoxemic respiratory failure due to COVID-19.

## Methods

### Study Design

We conducted an observational cohort study including all adult (≥18 years old) patients with laboratory-confirmed COVID-19, consecutively admitted in Evangelismos Hospital (Athens, Greece) between March 11, 2020 (the day of hospital admission of the first patient with COVID-19) and April 15, 2020. Evangelismos, the biggest tertiary-care hospital in Greece, serves as one of the three reference medical centers for treating patients with COVID-19 in Athens. In response to the pandemic, 72 ICU beds (from the initially available 30), never concomitantly occupied during the study period, were made available for inpatients.

### Compared Groups

Following collection of demographic and clinical data for the complete patient population through review of charts, patients with acute hypoxemic respiratory failure admitted in the ICU were categorized into the “early intubation” and the “delayed or no intubation” group. Acute hypoxemic respiratory failure was defined as the requirement for more than 5 L/min nasal oxygen (or Venturi mask more than 40%) to keep a pulse oximeter measured arterial blood oxygen saturation (SpO2) of equal to or more than 95%. “Delayed or no intubation” group consisted of patients receiving non-rebreather mask for equal to or more than 24 h or high-flow nasal oxygen for any period of time or non-invasive mechanical ventilation for any period of time in an attempt to avoid intubation. The remaining intubated patients comprised the “early intubation” group. The decision of early vs. delayed or no intubation rested with the treating clinicians. Clinicians of our department decided intubation in case of hemodynamic instability, altered mentation and respiratory distress (as evidenced by the usage of accessory respiratory muscles or inability to speak). Rather, hypoxemia without respiratory distress or dyspnea (i.e., the silent hypoxemia, which may be commonly seen of patients with COVID-19) was not usually considered enough to trigger intubation in accordance to relevant reports highlighting both the confounders affecting the quantification of hypoxemia and its association with the physiologic state of patients with COVID-19 (10).

### Study Outcomes

ICU-mortality, ventilator-free days and ICU-free days were the outcomes of the study. ICU-mortality was censored at 28 days after the occurrence of acute hypoxemic respiratory failure. Ventilator-free days were calculated starting at the first 24 continuous hours without invasive mechanical ventilation. The day of acute hypoxemic respiratory failure occurrence was considered as day 0 of the 28-day period for which ventilator-free days were calculated. Periods of extubation lasting for equal to or <48 h before re-intubation were not calculated in the sum of ventilator-free days (11). ICU-free days were calculated starting at the first 24 continuous hours outside the ICU in the post-ICU discharge period. The day of ICU admission was considered as day 0 of the 28-day period for which ICU-free days were calculated. Occurrence of septic shock (defined according to Sepsis-3) (12) and need of continuous renal replacement therapy also served as secondary outcomes of the study.

### Statistical Analysis

Study population included all patients treated during the study period. Continuous variables are presented as median and interquartile range (IQR). Mann-Whitney rank sum-test was used to compare continuous variables. Categorical variables are presented as number of patients (percentage). X^2^ or Fisher exact-test was used to compare categorical variables. A binary logistic regression analysis was carried out to isolate the contribution of early intubation and sex (independent variables) to mortality (categorical dependent variable). All statistical tests were 2-tailed and statistical significance was defined as *p* < 0.05. Statistical analyses were performed using SPSS software ver. 22.0 (IBM, Armonk, NY, USA).

## Results

**Figure 1:**
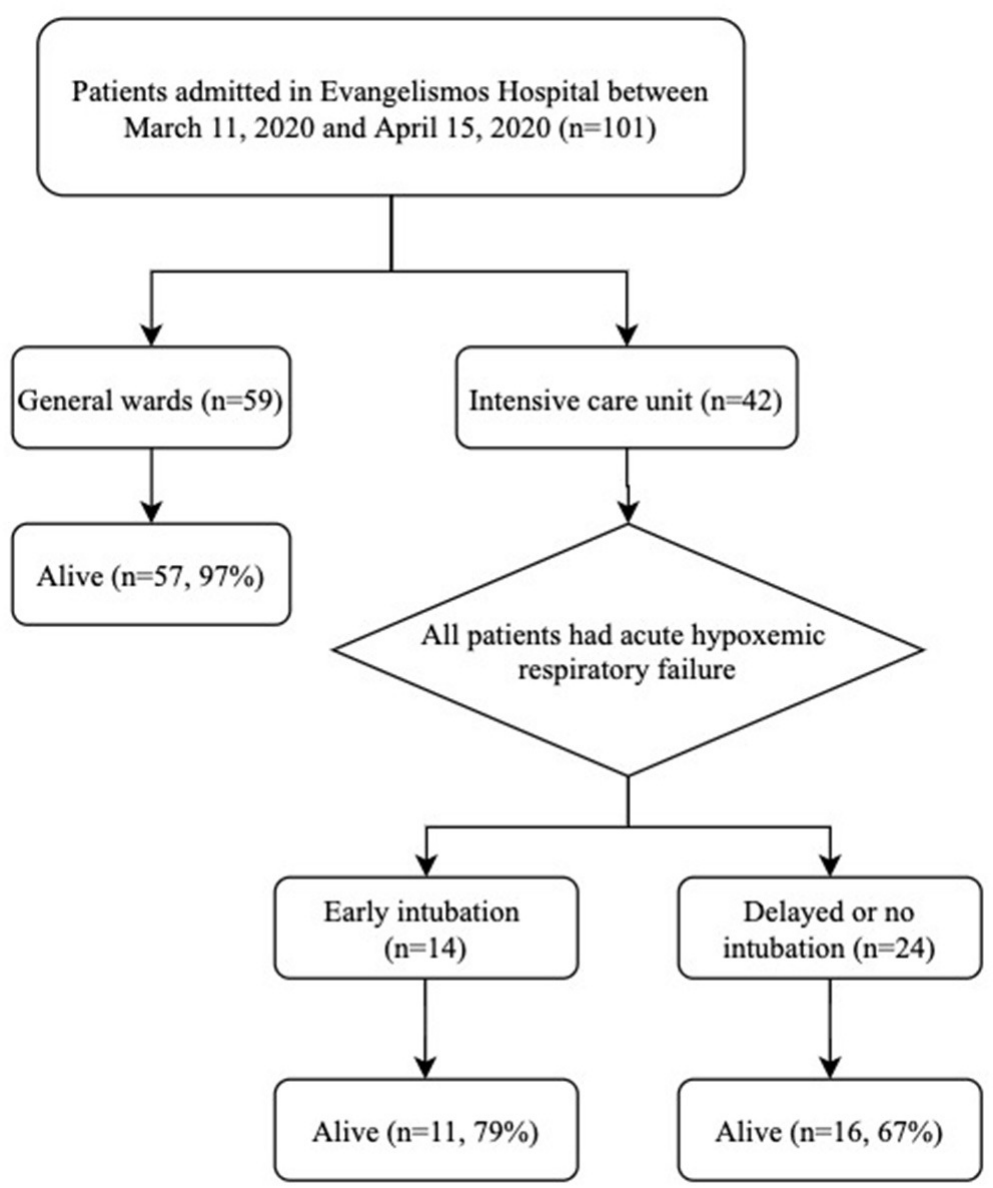
Patient flow diagram. Six patients admitted in the intensive care unit were not intubated and therefore were included in the delayed or no intubation group. Four patients (transferred intubated from another hospital) were not categorized into the early vs. delayed or no intubation group due to unavailability of relevant data.

During the study period, a total of 101 patients [37% female, median age 65 (IQR 53-73) years] were admitted in the hospital (Figure 1). Fifty-nine patients (58% of the entire cohort) were exclusively hospitalized in general wards. Their mortality rate was 3% (only two patients, who opted out ICU admission, died) and median length of stay was 7 days (IQR 5-13). None of the healthcare-workers of the hospital tested positive for COVID-19.

Table 1 summarizes the baseline characteristics and outcomes of 42 patients admitted in the ICU (all with acute hypoxemic respiratory failure) during the study period. The median time from hospital to ICU admission was 0 (IQR 0-3) days. Of those admitted in the ICU, 19% were female and 62% had at least one comorbidity. Their median age was 65 (IQR 58-71) years. None of those had a do-not-intubate order and 36 (86%) patients were indeed intubated. ICU-mortality among patients admitted in the ICU was 26%. Data for 13 of those patients have been included in a previous report focusing on the application of positive end-expiratory pressure (13).

**Table 1:** Baseline characteristics and outcomes of patients admitted in the intensive care unit.

	**All (*n* = 42)**	**Early intubation (*n* = 14)**	**Delayed or no intubation (*n* = 24)**	***p-*value**
Age, years (IQR)	65 (58–71)	63 (57–69)	64 (57–74)	0.68
Sex, female, *n* (%)	8 (19)	6 (43)	2 (8)	0.03
Race, *n* (%)				0.69
Caucasian	39 (93)	14 (100)	21 (85)	
Asian	2 (5)	0 (0)	2 (8)	
Middle Eastern	1 (2)	0 (0)	1 (4)	
Comorbidity, *n* (%)	26 (62)	8 (57)	15 (63)	0.74
Cardiovascular	20 (48)	7 (50)	11 (46)	0.80
Diabetes Mellitus	7 (17)	2 (14)	5 (21)	1
Chronic lung disease	4 (10)	1 (7)	3 (13)	1
Renal failure	1 (2)	0 (0)	1 (4)	1
Malignancy	5 (12)	0 (0)	4 (17)	0.27
SOFA score (IQR)	4 (4–6)	4 (4–5)	4 (4–6)	0.8
Respiratory	4 (3–4)	4 (4–4)	4 (3–4)	0.11
Coagulation	0 (0–0)	0 (0–0)	0 (0–1)	0.16
Hepatic	0 (0–0)	0 (0–0)	0 (0–0)	0.3
Neurologic	0 (0–0)	0 (0–0)	0 (0–0)	0.97
Cardiovascular	0 (0–0)	0 (0–0)	0 (0–0)	0.2
Renal	0 (0–0)	0 (0–0)	0 (0–0)	0.77
Usage of non-rebreather mask, *n* (%)	41 (98)	14 (100)	23 (96)	1
Usage of high-flow nasal oxygen, *n* (%)	11 (26)	0 (0)	11 (46)	0.003
Usage of non-invasive mechanical ventilation, *n* (%)	2 (5)	0 (0)	2 (8)	0.52
Lung mechanics at day of intubation, (IQR)				
Ppeak	NA	39 (36–41)	37 (32–42)	0.6
Pplateau	NA	28 (28–31)	28 (25–32)	0.45
PEEPtotal	NA	17 (13–19)	14 (11–19)	0.2
Pdriving	NA	13 (10–15)	13 (12–17)	0.27
Transferred intubated from another hospital, *n* (%)	19 (45)	12 (86)	3 (13)	<0.001
**Outcomes within 28 days**				
Intubation, *n* (%)	36 (86)	14 (100)	18 (75)	0.06
Intubation outside ICU, *n* (%)	21 (50)	12 (86)	6 (25)	0.004
Septic shock, *n* (%)	18 (43)	6 (43)	11 (46)	1
Continuous renal replacement therapy, *n* (%)	17 (41)	4 (29)	12 (50)	0.19
Ventilator-free days, days (IQR)	NA	3 (0–17)	2 (1–13)	0.57
ICU-free days, days (IQR)	0 (0–15)	0 (0–16)	0 (0–12)	0.59
Time from acute respiratory failure to ICU admission, days (IQR)	1 (0–1)	1 (0–1)	1 (0–2)	0.87
ICU-mortality, *n* (%)	11 (26)	3 (21)	8 (33)	0.48

*IQR, interquartile range; SOFA, sequential organ failure assessment; NA, not applicable; ICU, intensive care unit*.

*“Delayed or no intubation” group consisted of patients receiving non-rebreather mask for equal to or more than 24 h or high-flow nasal oxygen for any period of time or non-invasive mechanical ventilation for any period of time in an attempt to avoid intubation. The remaining intubated patients comprised the “early intubation” group*.

*Six patients admitted in the ICU were not intubated and therefore were included in the delayed or no intubation group. Four patients (transferred intubated from another hospital) were not categorized into the early vs. delayed or no intubation group due to unavailability of relevant data*.

*Cardiovascular comorbidities included congestive heart failure, hypertension, coronary artery disease, dyslipidemia and valvular dysfunction*.

Table 1 also summarizes the baseline characteristics and outcomes of patients undergoing early vs. delayed or no intubation. Four patients (all transferred intubated from another hospital) were not categorized into the early vs. delayed or no intubation group due to unavailability of relevant data. Baseline characteristics [including age, comorbidities and organ failure, as assessed by the Sequential Organ Failure Assessment (SOFA) scores, on the day of occurrence of acute hypoxemic respiratory failure] were comparable between the two groups with the exemption of sex. Regarding means of oxygenation, non-rebreather mask was used by all but one patient (who belonged in the delayed or no intubation group), while high-flow nasal oxygen and non-invasive mechanical ventilation was used by 11 and two patients, respectively. Regarding outcomes, early intubation was not associated with higher ICU-mortality, (21 vs. 33%), fewer ventilator-free days (3 vs. 2 days) or fewer ICU-free days (0 vs. 0 days) than delayed or no intubation. Early intubation was associated with lower (albeit statistically non-significant) need for continuous renal replacement therapy (29 vs. 50%) than delayed or no intubation. The above findings persisted when comparing the baseline characteristics and outcomes of patients undergoing early vs. delayed intubation, i.e., after exclusion of six ICU patients who were not intubated (Supplementary Table). Time from acute respiratory failure to intubation was shorter for the early intubation compared to the delayed intubation group (0 vs. 2 days) (Supplementary Table).

Early intubation (as opposed to delayed or no intubation) was not associated with mortality even after adjustment for sex (i.e., a baseline characteristic which differed between the two groups).

## Discussion

We found that approximately one-fourth of patients admitted in the ICU with acute hypoxemic respiratory failure due to laboratory confirmed COVID-19 in Athens, Greece died during their ICU stay. We also found that early intubation was not associated with worse clinical outcomes, such as mortality, ICU-free days and ventilator-free days, compared to delayed or no intubation among those patients.

The observed mortality rate of 26% for patients with COVID-19 admitted in our ICU seems lower than the mortality rates of 62 and 51% reported by early studies from Wuhan, China and Washington State, USA, respectively (14, 15). Although the latter mortality rates might be exaggerated (16) and subsequent studies reported outcomes similar to ours (17), this finding is intriguing. It could be explained by the fact that the health-care system of Greece was not substantially burdened throughout the course of the COVID-19 outbreak. Indeed, a substantially burdened health-care system might lead to worse outcomes (9). Thus, our finding regarding mortality rate may highlight the beneficial effect of protecting health-care care systems (e.g., through early flattening of the epidemiological curve) from overwhelming on outcomes of critically ill patients with COVID-19.

We found that a strategy of early intubation, as opposed to delayed or no intubation, was not associated with worse clinical outcomes, such as mortality, ventilator-free days and ICU-free days. Rather, it seems that the difference in terms of mortality (early: 21% vs. delayed or no: 33%) and ventilator-free days (early: 3 vs. delayed or no: 2 days) was in favor of the early than the delayed or no intubation strategy. Especially, the observed 12% absolute reduction in mortality with early intubation (which did not reach statistical significance, presumably due to small sample size) may indeed be clinically significant. This finding does not seem to justify the hesitance of clinicians to perform early intubation in concern that it may inadvertently lead to otherwise preventable intubations. In the light of our finding that an early intubation strategy might not be associated with increased mortality and morbidity, one could advocate this approach when taking into consideration its potential benefit of reduced viral aerosolization. To this end, early intubation and avoidance of prolonged use of high-flow nasal oxygen and non-invasive mechanical ventilation (although a short trial should not be precluded) has been advised by various societies' guidelines to prevent risks for patients and healthcare workers (18, 19).

In addition to its usage as an infection control measure, early intubation could also serve as a means to prevent both emergent intubation and patient self-inflicting lung injury. Regarding emergent intubation, its avoidance could improve outcomes, including mortality (20), by reducing incidence of hypoxemia (21). Regarding patient self-inflicting lung injury, its prevention and the subsequent pulmonary-renal crosstalk (with or without the effect of intrathoracic pressures and positive end-expiratory pressure) might explain our finding that need for continuous renal replacement therapy was lower (albeit statistically non-significant) in the early vs. the delayed or no intubation group (29 vs. 50%) (22, 23). The latter finding could also be explained by the relative dehydration of patients struggling to maintain normoxemia and avoid intubation through the prolonged usage of non-rebreather mask or high-flow nasal oxygen or non-invasive mechanical ventilation.

Our study has limitations. Firstly, although we included all consecutive patients admitted in our hospital, our retrospective single-center study still has a moderate sample size. However, this is the case for several other studies involving critically ill patients with COVID-19 (17, 24). Also, the moderate sample size is the fortunate outcome of the early flattening of the epidemiological curve in Greece and eventually the reason we were able to estimate the effect of early vs. delayed or no intubation on outcomes of patients with COVID-19 without the major confounding factor of the shortage of ventilators. Secondly, similar to the vast majority of studies in the field of COVID-19 (1, 8, 25), our study is observational and therefore subject to confounding. Even though there was no difference at baseline between the compared groups in terms of variables known to affect prognosis of patients with COVID-19, such as age, comorbidities and severity of illness (as assessed by SOFA) (1), we cannot preclude potential residual confounding, which could only be eliminated if the study was designed as a randomized controlled trial. Besides, our main finding persisted even after adjusting for sex. Thirdly, although we presented data on pulmonary and circulatory SOFA at baseline (Table 1), we did not collect specific data on respiratory rate and heart rate, which could further inform readers regarding the decision for intubation. Finally, one could argue that the comparison of early vs. delayed intubation (i.e., after exclusion of ICU patients who were not intubated) should be the primary analysis of our report. To that end, we presented the aforementioned analysis in the Supplementary Table and found similar results as in our main analysis. Moreover, the fact that an early intubation strategy was not associated with worse outcomes even when the comparator included never intubated patients may further strengthens the findings of our study.

## Conclusions

The findings of our study suggest that early intubation, as opposed to delayed or no intubation, may not be associated with worse outcomes among critically ill patients with COVID-19. Given the observed lack of a negative effect of early intubation on mortality and morbidity of critically ill patients, such a therapeutic approach could be considered to avoid viral cross-contamination and to prevent self-inflicting lung injury. Thus, our study may justify further research with a prospective, randomized controlled trial.

## Data Availability Statement

The raw data supporting the conclusions of this article will be made available by the authors, without undue reservation.

## Ethics Statement

The studies involving human participants were reviewed and approved by The Institutional Review Board of Evangelismos approved of this study (#212, 2020) and waived the need for informed patient consent. Written informed consent for participation was not required for this study in accordance with the national legislation and the institutional requirements.

## Author Contributions

IS, EX, CR, and SZ study concept and design. IS, TN, DZ, and EM acquisition of data. EX and IS first drafting of the manuscript. EX statistical analysis. IS and SZ obtained funding and study supervision. AK and SZ administrative, technical, and material support. IS had full access to all the data in the study, takes responsibility for the integrity of the data and the accuracy of the data analysis, and data access and responsibility. All authors read and approved the final manuscript, analysis and interpretation of data, and critical revision of the manuscript for important intellectual content.

## Conflict of Interest

The authors declare that the research was conducted in the absence of any commercial or financial relationships that could be construed as a potential conflict of interest.

## Abbreviations

COVID-19: coronavirus disease 2019
ICU: intensive care unit
IQR: interquartile range
SOFA: sequential organ failure assessment.

## References

[1] Zhou F Yu T Du R Fan G Liu Y Liu Z Clinical course and risk factors for mortality of adult inpatients with COVID-19 in Wuhan, China: a retrospective cohort study. Lancet. (2020) 395:1054–62. 10.1016/S0140-6736(20)30566-3 32171076 PMC7270627

[2] Vetter P Vu DL L'Huillier AG Schibler M Kaiser L Jacquerioz F. Clinical features of covid-19. BMJ. (2020) 369:m1470. 10.1136/bmj.m1470 32303495

[3] Goyal P Choi JJ Pinheiro LC Schenck EJ Chen R Jabri A. Clinical characteristics of covid-19 in New York city. N Engl J Med. (2020) 382:2372–4. 10.1056/NEJMc2010419 32302078 PMC7182018

[4] Gattinoni L Chiumello D Rossi S. COVID-19 pneumonia: ARDS or not? Crit Care. (2020) 24:154. 10.1186/s13054-020-02880-z 32299472 PMC7160817

[5] Tran K Cimon K Severn M Pessoa-Silva CL Conly J. Aerosol generating procedures and risk of transmission of acute respiratory infections to healthcare workers: a systematic review. PLoS ONE. (2012) 7:e35797. 10.1371/journal.pone.0035797 22563403 PMC3338532

[6] Tobin MJ Laghi F Jubran A. Caution about early intubation and mechanical ventilation in COVID-19. Ann Intensive Care. (2020) 10:78. 10.1186/s13613-020-00692-6 32519064 PMC7281696

[7] Tobin MJ Laghi F Jubran A. P-SILI is not justification for intubation of COVID-19 patients. Ann Intensive Care. (2020) 10:105. 10.1186/s13613-020-00724-1 32748116 PMC7397710

[8] Grasselli G Zangrillo A Zanella A Antonelli M Cabrini L Castelli A. Baseline characteristics and outcomes of 1591 patients infected with SARS-CoV-2 admitted to ICUs of the Lombardy Region, Italy. JAMA. (2020) 323:1574–81. 10.1001/jama.2020.5394 32250385 PMC7136855

[9] Ji Y Ma Z Peppelenbosch MP Pan Q. Potential association between COVID-19 mortality and health-care resource availability. Lancet Global Health. (2020) 8:e480. 10.1016/S2214-109X(20)30068-1 32109372 PMC7128131

[10] Tobin MJ Laghi F Jubran A. Why COVID-19 silent hypoxemia is baffling to physicians. Am J Respir Crit Care Med. (2020) 202:356–60. 10.1164/rccm.202006-2157CP 32539537 PMC7397783

[11] Finkelsztein EJ Jones DS Ma KC Pabón MA Delgado T Nakahira K. Comparison of qSOFA and SIRS for predicting adverse outcomes of patients with suspicion of sepsis outside the intensive care unit. Crit Care. (2017) 21:73. 10.1186/s13054-017-1658-5 28342442 PMC5366240

[12] Singer M Deutschman CS Seymour CW Shankar-Hari M Annane D Bauer M The third international consensus definitions for sepsis and septic shock (Sepsis-3). JAMA. (2016) 315:801–10. 10.1001/jama.2016.0287 26903338 PMC4968574

[13] Tsolaki V Siempos I Magira E Kokkoris S Zakynthinos GE Zakynthinos S. PEEP levels in COVID-19 pneumonia. Crit Care. (2020) 24:303. 10.1186/s13054-020-03049-4 32505186 PMC7275848

[14] Yang X Yu Y Xu J Shu H Xia J Liu H. Clinical course and outcomes of critically ill patients with SARS-CoV-2 pneumonia in Wuhan, China: a single-centered, retrospective, observational study. Lancet Respir Med. (2020) 8:475–81. 10.1016/S2213-2600(20)30079-5 32105632 PMC7102538

[15] Arentz M Yim E Klaff L Lokhandwala S Riedo FX Chong M. Characteristics and outcomes of 21 critically Ill patients with COVID-19 in Washington State. JAMA. (2020) 323:1612–4. 10.1001/jama.2020.4326 32191259 PMC7082763

[16] Weiss P Murdoch DR. Clinical course and mortality risk of severe COVID-19. Lancet. (2020) 395:1014–5. 10.1016/S0140-6736(20)30633-4 32197108 PMC7138151

[17] Ziehr DR Alladina J Petri CR Maley JH Moskowitz A Medoff BD. Respiratory pathophysiology of mechanically ventilated patients with COVID-19: a cohort study. Am J Respir Crit Care Med. (2020) 201:1560–4. 10.1164/rccm.202004-1163LE 32348678 PMC7301734

[18] Brewster DJ Chrimes N Do TB Fraser K Groombridge CJ Higgs A. Consensus statement: Safe Airway Society principles of airway management and tracheal intubation specific to the COVID-19 adult patient group. Med J Aust. (2020) 212:472–81. 10.5694/mja2.50598 32356900 PMC7267410

[19] Sorbello M El-Boghdadly K Giacinto ID Cataldo R Esposito C Falcetta S. The Italian coronavirus disease 2019 outbreak: recommendations from clinical practice. Anaesthesia. (2020) 75:724–32. 10.1111/anae.15049 32221973

[20] Meng L Qiu H Wan L Ai Y Xue Z Guo Q. Intubation and ventilation amid the COVID-19 outbreak. Anesthesiology. (2020) 132:1317–32. 10.1097/ALN.0000000000003296 32195705 PMC7155908

[21] Yao W Wang T Jiang B Gao F Wang L Zheng H. Emergency tracheal intubation in 202 patients with COVID-19 in Wuhan, China: lessons learnt and international expert recommendations. Br J Anaesth. (2020) 125:e28–37. 10.1016/j.bja.2020.03.026 32312571 PMC7151238

[22] Domenech P Perez T Saldarini A Uad P Musso CG. Kidney-lung pathophysiological crosstalk: its characteristics and importance. Int Urol Nephrol. (2017) 49:1211–5. 10.1007/s11255-017-1585-z 28401379

[23] Anile A Castiglione G Zangara C Calabrò C Vaccaro M Sorbello M. COVID: the new ultrasound alphabet in SARS-CoV-2 era. Anesth Analg. (2020) 131:e232–34. 10.1213/ANE.0000000000005142 33094983 PMC7389191

[24] Elharrar X Trigui Y Dols A-M Touchon F Martinez S Prud'homme E. Use of prone positioning in nonintubated patients with COVID-19 and hypoxemic acute respiratory failure. JAMA. (2020) 323:2336–8. 10.1001/jama.2020.8255 32412581 PMC7229532

[25] Guan W Ni Z Hu Y Liang W Ou C He J. Clinical characteristics of coronavirus disease 2019 in China. N Engl J Med. (2020) 382:1708–20. 10.1056/NEJMoa2002032 32109013 PMC7092819

