# Supplementary material for: Effect of Early vs. Delayed or No Intubation on Clinical Outcomes of Patients With COVID-19: An Observational Study
Source: Front Med (Lausanne). 2020 Dec 23;7:614152. doi: 10.3389/fmed.2020.614152 (PMC7785771; doi:10.3389/fmed.2020.614152)
Supplement: Supplementary file 1 [file Table_1.docx]

| **Supplemental Table. Baseline characteristics and outcomes of intubated patients admitted in the intensive care unit.** | | | | |
| --- | --- | --- | --- | --- |
|  | **All intubated**  **(n=36)** | **Early intubation (n=14)** | **Delayed intubation**  **(n=18)** | **p value** |
| Age, years (IQR) | 66 (62-75) | 63 (57-69) | 68 (58-75) | 0.37 |
| Sex, female, n (%) | 8 (22) | 6 (43) | 2 (11) | 0.1 |
| Race, n (%) |  |  |  | 1 |
| Caucasian | 35 (97) | 14 (100) | 17 (94) |  |
| Asian | 1 (3) | 0 (0) | 1 (6) |  |
| Middle Eastern | 0 (0) | 0 (0) | 0 (0) |  |
| Comorbidity, n (%) | 23 (64) | 8 (57) | 12 (67) | 0.58 |
| Cardiovascular | 18 (20) | 7 (50) | 9 (50) | 1 |
| Diabetes Mellitus | 6 (17) | 2 (14) | 4 (22) | 0.67 |
| Chronic lung disease | 2 (6) | 1 (7) | 1 (6) | 1 |
| Renal failure | 1 (3) | 0 (0) | 1 (6) | 1 |
| Malignancy | 5 (14) | 0 (0) | 4 (22) | 0.11 |
| SOFA score (IQR) | 6 (4-7) | 4 (4-5) | 5 (4-6) | 0.4 |
| Respiratory | 4 (4-4) | 4 (4-4) | 4 (3-4) | 0.32 |
| Coagulation | 0 (0-1) | 0 (0-0) | 0 (0-0) | 0.22 |
| Hepatic | 0 (0-1) | 0 (0-0) | 0 (0-0) | 0.28 |
| Neurologic | 0 (0-1) | 0 (0-0) | 0 (0-1) | 0.72 |
| Cardiovascular | 0 (0-0) | 0 (0-0) | 0 (0-0) | 0.27 |
| Renal | 0 (0-1) | 0 (0-0) | 0 (0-0) | 0.5 |
| Usage of non-rebreather mask, n (%) | 34 (94) | 14 (100) | 18 (100) | 1 |
| Usage of high-flow nasal oxygen, n (%) | 9 (25) | 0 (0) | 9 (50) | 0.002 |
| Usage of non-invasive mechanical ventilation, n (%) | 2 (6) | 0 (0) | 2 (11) | 0.49 |
| Lung mechanics at day of intubation (IQR) |  |  |  |  |
| Ppeak | 37 (35-40) | 39 (36-41) | 37 (32-42) | 0.6 |
| Pplateau | 28 (25-30) | 28 (28-31) | 28 (25-32) | 0.45 |
| PEEPtotal | 14 (10-17) | 17 (13-19) | 14 (11-19) | 0.21 |
| Pdriving | 15 (20-17) | 13 (10-15) | 13 (12-17) | 0.27 |
| Transferred intubated from another hospital, n (%) | 19 (53) | 12 (86) | 3 (17) | <0.001 |
| **Outcomes within 28 days** |  |  |  |  |
| Intubation, n (%) | 36 (100) | 14 (100) | 18 (100) | 1 |
| Intubation outside ICU,  n (%) | 21 (58) | 12 (86) | 6 (33) | 0.003 |
| Septic shock, n (%) | 18 (50) | 6 (43) | 11 (61) | 0.3 |
| Continuous renal replacement therapy, n (%) | 17 (47) | 4 (29) | 12 (67) | 0.07 |
| Ventilator-free days, days (IQR) | 1 (0-2) | 3 (0-17) | 2 (1-13) | 0.57 |
| ICU-free days, days (IQR) | 0 (0-0) | 0 (0-16) | 0 (0-4) | 0.39 |
| Time from acute respiratory failure to intubation, days (IQR) | 1 (0-2) | 0 (0-0) | 2 (1-3) | <0.001 |
| Time from acute respiratory failure to ICU admission, days (IQR) | 1 (0-1) | 1 (0-1) | 1 (0-2) | 0.44 |
| ICU-mortality, n (%) | 11 (30) | 3 (21) | 8 (44) | 0.26 |
| *Abbreviations:* IQR: interquartile range, SOFA: sequential organ failure assessment, NA: not applicable, ICU: intensive care unit | | | | |
